# Biodegradable pins for lateral condylar fracture of the humerus with an early delayed presentation in children: a retrospective study of biodegradable pin vs. Kirschner wire

**DOI:** 10.1186/s12891-020-03774-5

**Published:** 2020-11-11

**Authors:** Jin Li, Saroj Rai, Yun Gao, Renhao Ze, Xin Tang, Ruikang Liu, Pan Hong

**Affiliations:** 1 Department of Orthopaedic Surgery, Union Hospital, Tongji Medical College, Huazhong University of Science and Technology, Wuhan, 430022 China; 2 Department of Orthopaedics and Trauma Surgery, National Trauma Center, National Academy of Medical Sciences, Mahankal, Kathmandu, Nepal; 3 Department of Orthopaedic Surgery, Zhuhai Center for Maternal and Child Health Care, Zhuhai, China; 4 First School of Clinical Medicine, Tongji Medical College, Huazhong University of Science and Technology, Wuhan, China

**Keywords:** Delay, Lateral condylar fracture, Children, Biodegradable pin

## Abstract

**Background:**

The clinical outcome of open reduction and internal fixation (ORIF) for delayed lateral condylar fracture of the humerus (LCFH) varies in different studies, but ORIF for LCFH with an early-delayed presentation usually resulted in significant improvement of elbow function. Early delayed presentation is defined as a period of 3 to 12 weeks from the injury. This study aims to compare the clinical outcomes of biodegradable pin (BP) vs. Kirschner wire (KW) in the treatment of LCFH with an early delayed presentation.

**Methods:**

LCFH with an early-delayed presentation treated with KW or BP were retrospectively reviewed in our hospital. The patients were divided into two groups KW (*n* = 17) and BP group (*n* = 26). Baseline information, including sex, age, operative side, duration from injury to surgery, and implant choice, was reviewed. Radiographs and medical records were collected from the Hospital Database.

**Results:**

In all, 17 patients (male/female, 9/8) in KW and 26 patients (male/female,13/13) in the BP group were included. The age showed no statistically significant difference between the KW (52.3 ± 10.2, month) and the BP (56.1 ± 10.7, month), (*P* = 0.258). At the last follow-up, there existed no statistically significant difference between the two groups concerning Baumann’s angle (*P* = 0.272) and carrying angle (*P* = 0.911). The MEPS at the last follow-up was better in the KW group (91.1 ± 2.7) than the BP group (89.2 ± 3.0), (*P* = 0.048). There was no case of nonunion or malunion in both groups. The incidence of fishtail deformity was (8/17, 47.1%) in KW and (13/26, 50%) in the BP group. The incidence of lateral prominence was (5/17, 29.4%) in the KW and (7/26, 26.9%) in the BP group. Furthermore, the incidence of implant prominence was higher in KW (12/17, 70.6%) than BP (0) (*P* <  0.001).

**Conclusion:**

Open reduction and internal fixation for LCFH with an early-delayed presentation produced satisfactory outcomes. Biodegradable pin is a good alternative to Kirschner wire, with comparable clinical outcomes.

## Background

Lateral condylar fracture of the humerus (LCFH) is a common elbow injury in children [1]. Early diagnosis with appropriate treatment normally yields satisfactory outcomes [2]. However, if the diagnosis is delayed or the reduction is lost after initial conservative treatment, malunion or nonunion usually occurs. Patients suffering from malunion or nonunion usually present with persistent pain, decreased range of movement (ROM) of the elbow joint, cubitus valgus deformity, and delayed ulnar nerve palsy [3–5]. The current treatment method for neglected or delayed LCFH is controversial, which ranges from observation with periodical follow-up to open reduction and internal fixation (ORIF) [3, 4, 6]. The clinical outcomes following ORIF of the LCHF have been reported differently in different studies [3–5, 7], however, ORIF for an early-delayed presentation usually resulted in significant improvement of elbow function [3].

Utilization of Kirschner wire (KW) for the fixation of LCHF with an early-delayed presentation is a cost-effective choice [3, 8, 9], so it is our preferred choice before the introduction of biodegradable pin (BP). Early-delayed presentation is defined as a period between 3 to 12 weeks from injury. This study aims to compare the clinical outcomes of BP with KW in the treatment of LCFH with an early-delayed presentation.

## Methods

LCFH with an early-delayed presentation treated with KW or BP from January 2010 to January 2016, at Wuhan union hospital, Tongji Medical College, Huazhong University of Science and Technology were retrospectively reviewed. This study was approved by the Ethics Committee of Tongji Medical College, Huazhong University of Science and Technology (IORG No: IORG0003571) on November 20, 2019. Written consent was obtained from the patient’s legal guardians.

Exclusion criteria are (1) patients with incomplete clinical data or radiographs; (2) open or pathological fracture; (3) presence of concomitant injuries of the elbow (fractures or dislocation); (4) duration of injury to surgery, less than 3 weeks or more than 12 weeks; (5) follow-up period of less than 24 months; (6) patients older than 14 years at the time of surgery.

BP is expensive, and not covered by basic medical insurance in our province. The pros and cons of two fixation materials were explained to the parents, and let them choose accordingly. The patients were divided into two groups KW (*n* = 17) and BP group (*n* = 26). Baseline information, including sex, age, operative side, duration from injury to surgery, and implant choice, was reviewed. Radiographs and medical records were collected from the Hospital Database. Fracture displacement was evaluated according to the Song classification [10]. The function of the elbow joint was evaluated according to the Mayo elbow performance score (MEPS) [11]. Complications, including infection, avascular necrosis (AVN), stiffness of elbow joint, and implant prominence and exposure, were carefully recorded.

### Biodegradable pins

Biodegradable Pins are made of a blend of L-lactide, D, L-lactide and trimethylene carbonate (TMC), with a diameter of 2.0 mm and length of 5.0 cm.

### Surgical technique

**Fig. 1:**
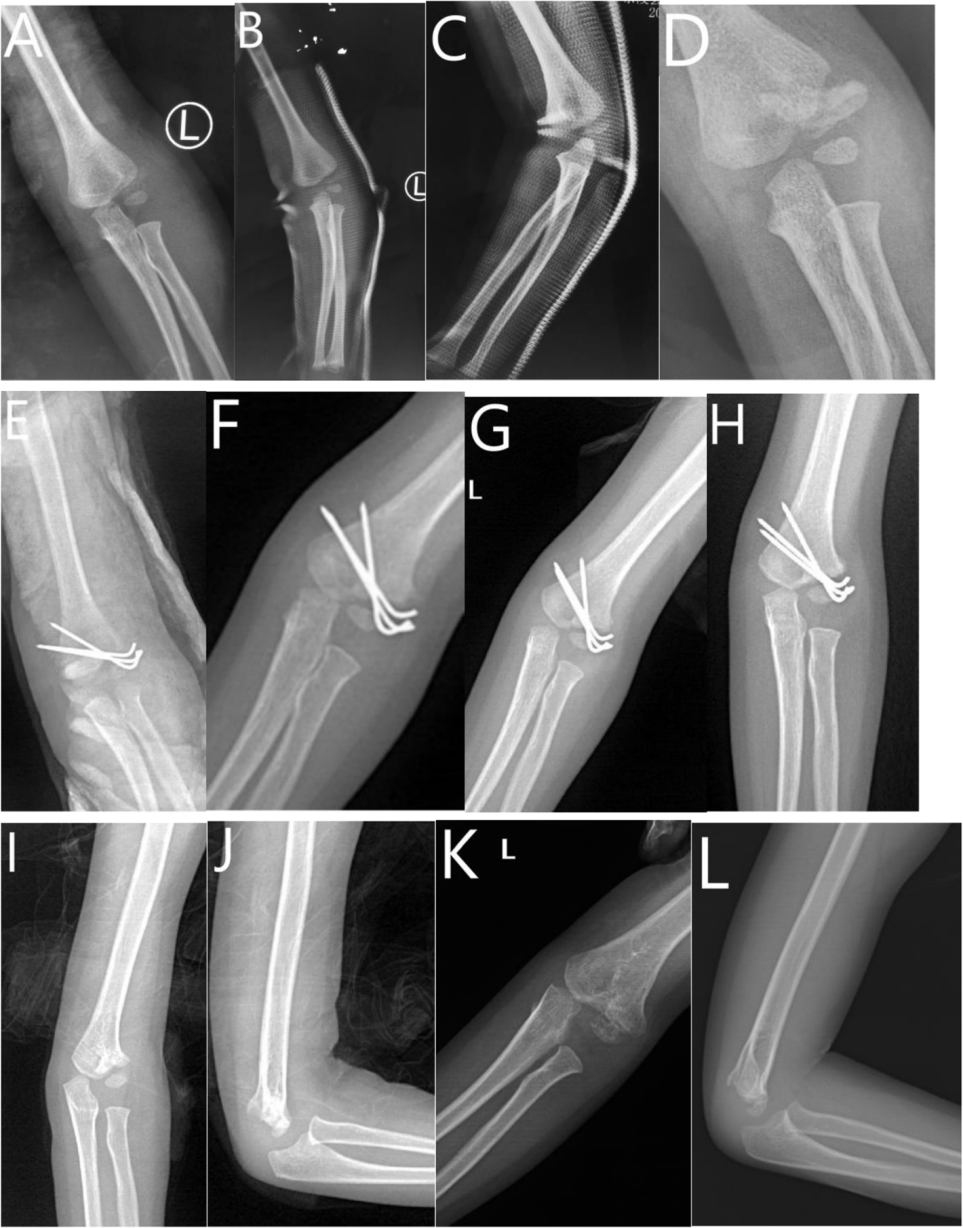
Three year-old boy of left delayed LCF treated with ORIF. **a** AP view of elbow at injury. **b** AP view of elbow 2 weeks after injury. **c** Lateral view of elbow 2 weeks after injury. **d** AP view of elbow 5 weeks after injury. **e** AP view of elbow after surgery. **f** AP view of elbow at 5th week follow-up. **g** AP view of elbow at 2nd month follow-up. **h** AP view of elbow at 4th month follow-up. **i** AP view of elbow at 9th month follow-up. **j** Lateral view of elbow at 9th month follow-up. **k** AP view of elbow at 25th month follow-up. **l** Lateral view of elbow at 25th month follow-up

Under general anesthesia, a 4–6 cm incision was made on the lateral aspect of the elbow, exposing the lateral and anterior capitulum. After removing the scar tissue from the fracture site, the fragment was reduced and fixated by 2–3 wires (diameter, 1.6 mm or 2 mm) under direct visualization of the articular surface. During the surgery, meticulous care was taken to preserve the soft tissue attachment of the lateral condylar fragment posteriorly. The KWs were routinely buried under the skin at our institute (Fig. 1).

**Fig. 2:**
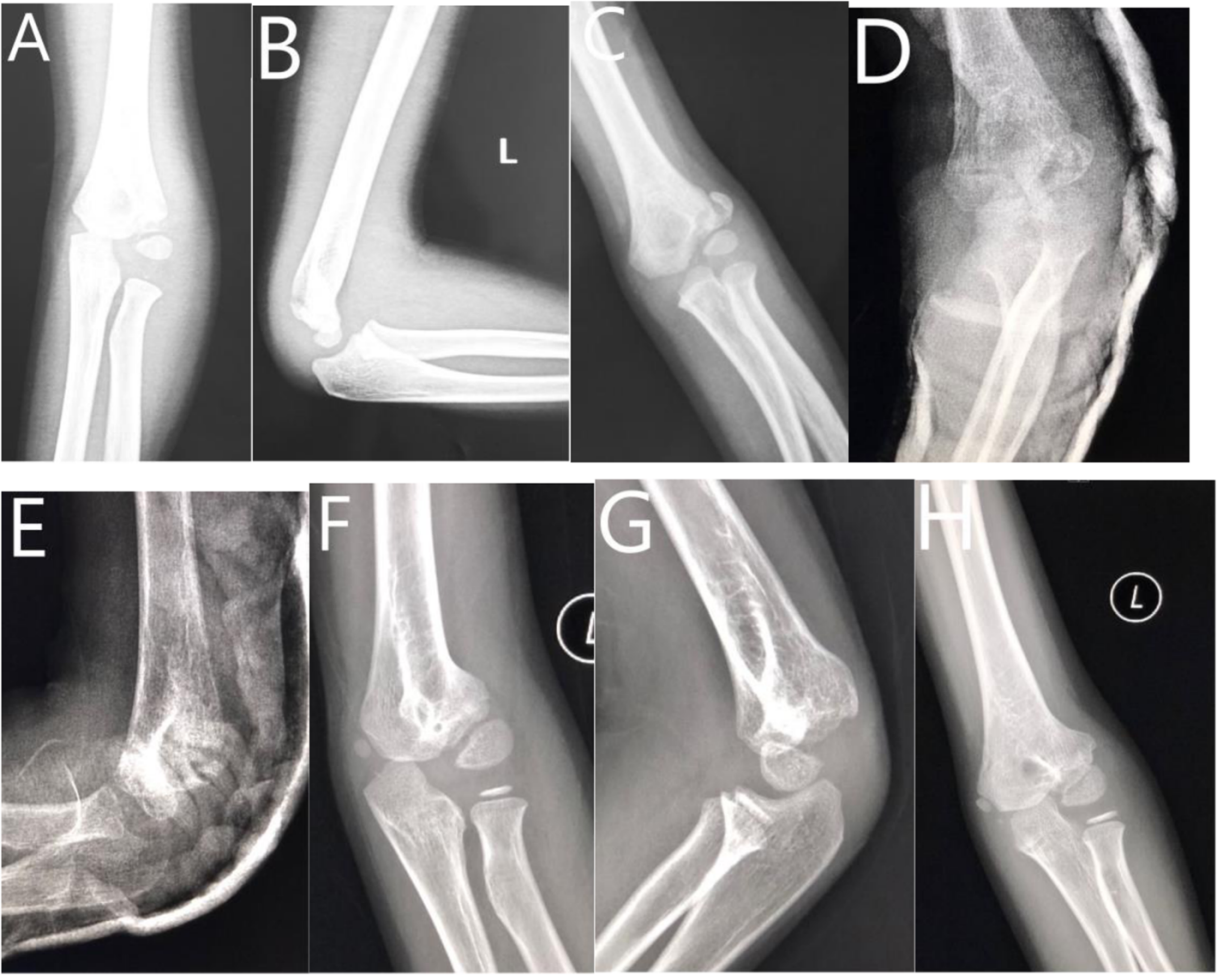
Six-year-old boy of left delayed lateral condylar fracture treated with BP. **a** AP view of elbow joint at the time of injury. **b** Lateral view of elbow joint at the time of injury. **c** AP view of elbow joint at 6th week follow-up after initial injury. **d** AP view of elbow joint after surgery. **e** Lateral view of elbow joint after surgery. **f** AP view of elbow joint at 16th month follow-up after surgery. **g** Lateral view of elbow joint at 16th month follow-up after surgery. **h** AP view of elbow joint at 25th month follow-up after surgery

In the BP group, the length of KW inside the bony surface was measured and replaced by the BP of the same size and length. Besides, a “figure of 8-band wiring” using a 1–0 absorbable suture was routinely performed to strengthen the stability (Fig. 2).

After the surgery, the arm was immobilized in the long-arm slab in a functional position for 3–5 weeks.

### Postoperative care and follow-up

The slab was removed at the out-patient visit about 3–5 weeks after the surgery and was dependent on the callus formation on the radiograph. The active exercise was encouraged after the slab removal. In the patients of the KW group, the removal of implants was scheduled after full consolidation of the fracture on the radiograph.

### Statistical analysis

SPSS statistical package program (SPSS 19.0 version; SPSS Inc., Chicago, Illinois, USA) was used for statistical analysis. The categorical data were analyzed using the Chi-square (χ^2^) test, and the continuous data were analyzed using Student’s t-test. Fisher exact test was used under those circumstances with fewer subjects in groups of interest. Data are presented as mean ± SD (range), median (range) or n (%). *P* <  0.05 was considered significantly different.

## Results

As shown in Table 1, 17 patients (male/female, 9/8) in KW and 26 patients (male/female,13/13) in the BP group were included in this study. The age showed no statistically significant difference between the KW (52.3 ± 10.2, month) and the BP (56.1 ± 10.7, month), (*P* = 0.258). There was no statistically significant difference between the two groups concerning other parameters such as sex, fracture side, and Song classification. But the duration from injury to surgery is longer in the BP group (55.7 ± 14.5, day) than the KW group (45.1 ± 15.5, day), (*P* = 0.034).

**Table 1:** Demographics of the patients

Parameters	KW (*n* = 17)	BP(*n* = 26)	*P* value
Age (months)	52.3 ± 10.2	56.1 ± 10.7	0.258
Sex (male/female)	9/8	13/13	0.852
Side (left/right)	8/9	13/13	0.852
From injury to surgery (d)	45.1 ± 15.5	55.7 ± 14.5	0.034^*^
Song Classification			
Type III	11	18	0.142
Type IV	6	8	

*< 0.05

As shown in Table 2, there was no significant difference between KW and BP on preoperative MEPS (*P* = 0.409). At the last follow-up, there existed no significant difference between the two groups concerning Baumann’s angle (*P* = 0.272) and carrying angle (*P* = 0.911). The MEPS at the last follow-up was better in the KW group (91.1 ± 2.7) than the BP group (89.2 ± 3.0), (*P* = 0.048).

**Table 2:** Clinical outcome of the patients

Clinical outcomes	KW (*n* = 17)	BP (*n* = 26)	*P* value
Baumann’s angle	19.2 ± 4.7	17.6 ± 4.5	0.272
Carrying angle	5.2 ± 3.2	5.3 ± 2.8	0.911
Preoperative MEPS	64.6 ± 3.1	65.4 ± 2.6	0.409
MEPS at last follow-up	91.1 ± 2.7	89.2 ± 3.0	0.048*

*< 0.05

As shown in Table 3, there was no case of nonunion or malunion observed in both groups. The incidence of implant exposure was higher in KW (2/17, 11.8%) than BP (0). The incidence of superficial infection is higher in KW (3/17, 17.6%) than BP (0), but there was no case of revision for infection. Besides, there was no case of unresolved stiffness, pain, and AVN in both groups. The incidence of fishtail deformity was (8/17, 47.1%) in KW and (13/26, 50%) in the BP group. The incidence of lateral prominence was (5/17, 29.4%) in KW and (7/26, 26.9%) in the BP group. Furthermore, the incidence of implant prominence was higher in KW (12/17, 70.6%) than BP (0) (*P* < 0.001).

**Table 3:** Complications of the patients

Complications	KW (*n* = 17)	BP (*n* = 26)	*P* value
Nonunion	0	0	1
Malunion	0	0	1
Exposure of implant	2 (11.8%)	0	0.076
Revision after infection	0	0	1
AVN	0	0	1
Unresolved stiffness	0	0	1
Fishtail deformity	8 (47.1%)	13 (50.0%)	0.852
Pain	0	0	1
Implant prominence	12 (70.6%)	0	< 0.001^*^
Lateral prominence	5 (29.4%)	7 (26.9%)	0.835
Superficial infection	3 (17.6%)	0	0.028^*^

^*^< 0.05

## Discussion

ORIF for LCF with an early-delayed presentation produced satisfactory clinical outcomes. BP requires no secondary surgery for hardware removal, with comparable clinical outcomes with KW.

Delayed LCFH in children is not common, and only a few case series have been reported [4, 5, 12, 13]. Besides, the optimal choice of treatment for this condition remains controversial [2, 3, 8]. The conservative method possibly results in poor clinical outcomes, including pain, instability of elbow joint, cubitus valgus deformity, and resultant ulnar nerve palsy [3–5, 12]. However, surgical intervention carries a potential risk of AVN, infection, stiffness of the joint, and growth disturbance of distal humerus [14, 15]. In patients with delayed presentation over 3 months, the morphology of the lateral condyle becomes unrecognizable and extensive dissection is usually required, which would compromise the vascular supply to lateral condyle fragment [16]. However, satisfactory outcomes have been reported in recent studies [4, 5, 14], especially in patients with an early-delayed presentation [3]. Therefore, ORIF was adopted at our institute for delayed LCFH.

Cannulated screw has been proposed for fresh LCFH [17, 18], and it has also been used in delayed cases [4, 5, 12]. ORIF with a screw delivers more inter-fragmentary compression across the fracture line and provides more stability [10]. However, it might not be practical, especially in younger children in whom the fracture fragment is relatively small and mostly cartilaginous. Besides, the metallic screw necessitates a second surgery for hardware removal. At our institute, the cannulated screw is usually used in delayed LCFH over 3 months. Before the introduction of BP, KW was our preferred choice for LCFH with an early-delayed presentation. There was also a report on the use of a single bioabsorbable screw for displaced LCFH [19], but the lateral condylar fragment in the younger children is too small for a 3.5 mm screw; besides, one screw is not able to resist the rotational force. BP has been used at our institute for fresh LCFH since 2008, and the clinical outcome was satisfactory [20], consistent with the previous study [21]. The advantages of biodegradable material for fracture fixation is retaining the stability of metallic hardware without the need for implant removal operation [19–21]. As shown in the result, the clinical outcome in the KW group was satisfactory, consistent with the previous report [3]. Therefore, BP was used for LCFH with an early-delayed presentation thereafter. To our knowledge, it is the first report of BP in delayed LCFH in children.

For delayed LCFH over 3 months, a cannulated screw combined with KW is our preferred choice, due to the better compression effect of the screw and possible bone defect after extensive dissection. Moreover, bone grafting was not necessary for our patients due to limited bone defects after reduction, consistent with previous reports [3, 22].

The clinical outcomes in both groups were satisfactory with a low rate of complications and were comparable with the outcomes of ORIF for fresh LCFH [23]. However, the incidence of implant prominence was found higher in the KW group, since the end of the KW was bent to be buried under the skin while the pins in the BP group was cut along the bony surface. In younger patients, the prominence might hinder functional training. However, the clinical outcomes at the last follow-up showed no significant difference between the two groups. Although whether KW should be buried remains controversial [24, 25], but the healing time of delayed LCFH is estimated to be longer than fresh fracture. Therefore, KW was buried under the skin to lower the incidence of pin tract related complications at our institute.

There was no case of nonunion or malunion in both groups, possibly because of limited dissection and careful preservation of soft tissue attachment. The incidence of fishtail deformity in both groups was not significantly different, but much higher than ORIF for fresh LCFH [26], partly due to the stripping of fibrous tissues around the fragment. The incidence of lateral prominence and superficial infection in our study is comparable to ORIF for fresh LCFH [2, 23].

There were certain limitations to this study. The sample size was relatively small because this condition was not common. We undertook a retrospective investigation; therefore, our findings should be interpreted with caution. The allocation process of patients to either the KW group or BP group partly depended on the preference of the surgeon in charge, and this strategy may cause allocation bias. Besides, the biodegradable pins were more expensive (500–600 US dollars for each pin) than K-wires (5–10 US dollars), and it was not covered by the basic medical insurance in our province. Cost-effective analysis remains to be investigated. In the future, prospective, well-designed randomized control trials will be required to validate the advantages of biodegradable material for the treatment of LCFH.

## Conclusion

Open reduction and internal fixation for LCFH with an early-delayed presentation produced satisfactory outcomes. Biodegradable pin is a good alternative to Kirschner wire, with comparable clinical outcomes.

## Data Availability

The data sets supporting the conclusion of this article are included within the article. Upon request, raw data can be provided by the corresponding author.

## Abbreviations

LCFH: Lateral condylar fracture of the humerus
KW: Kirschner wire
BP: Biodegradable pin
AVN: Avascular necrosis
ORIF: Open reduction and internal fixation
